# Integration of highly anisotropic multiferroic BaTiO_3_–Fe nanocomposite thin films on Si towards device applications†

**DOI:** 10.1039/d0na00405g

**Published:** 2020-07-21

**Authors:** Matias Kalaswad, Bruce Zhang, Xuejing Wang, Han Wang, Xingyao Gao, Haiyan Wang

**Affiliations:** School of Electrical and Computer Engineering, Purdue University West Lafayette Indiana 47907 USA hwang00@purdue.edu; School of Materials Engineering, Purdue University West Lafayette Indiana 47907 USA

## Abstract

Integration of highly anisotropic multiferroic thin films on silicon substrates is a critical step towards low-cost devices, especially high-speed and low-power consumption memories. In this work, an oxide–metal vertically aligned nanocomposite (VAN) platform has been used to successfully demonstrate self-assembled multiferroic BaTiO_3_–Fe (BTO–Fe) nanocomposite films with high structural anisotropy on Si substrates. The effects of various buffer layers on the crystallinity, microstructure, and physical properties of the BTO–Fe films have been explored. With an appropriate buffer layer design, *e.g.* SrTiO_3_/TiN bilayer buffer, the epitaxial quality of the BTO matrix and the anisotropy of the Fe nanopillars can be improved greatly, which in turn enhances the physical properties, including the ferromagnetic, ferroelectric, and optical response of the BTO–Fe thin films. This unique combination of properties integrated on Si offers a promising approach in the design of multifunctional nanocomposites for Si-based memories and optical devices.

## Abstract 2

Integration of highly anisotropic multiferroic thin films on silicon substrates is a critical step towards low-cost devices, especially high-speed and low-power consumption memories.

## Graphical abstract

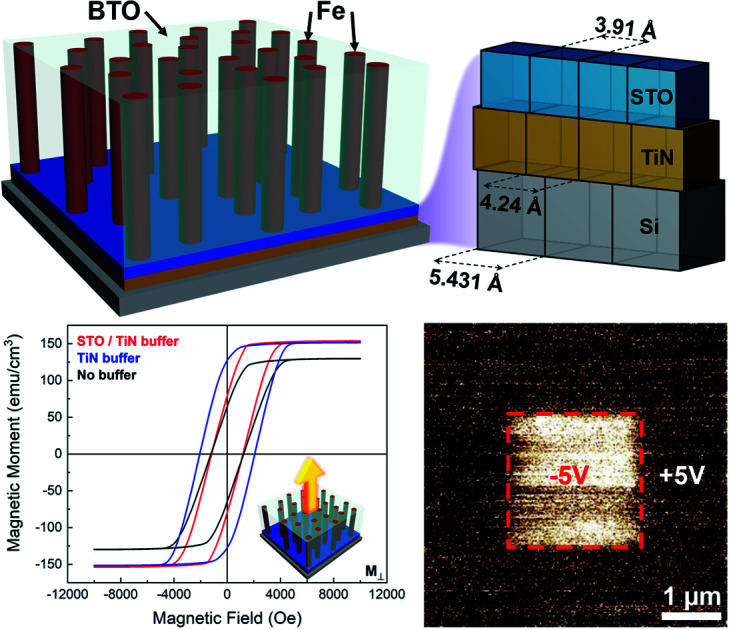

## Introduction

Multiferroic materials exhibit fascinating physical properties and offer a huge potential for device applications.^1–5^ By coupling at least two of the electrical, magnetic, or mechanical states, multiferroic materials can be used for a variety of applications including microwave phase shifters,^6,7^ multi-state memories,^8–10^ solar cells,^11–13^ and many others. For these reasons, there has been a great emphasis on discovering new multiferroic materials.^4,14^ However, the discovery of single-phase multiferroic films with strong, room-temperature physical properties has been proven to be difficult, largely due to the fact that the electronic configurations required for ferroelectric and ferromagnetic molecules are almost mutually exclusive.^14^ To overcome this issue, nanocomposite and multilayer films have been developed to couple materials with two different ferroic orders to produce a single multiferroic system.^15–18^ For example, the well-studied ferroelectric and piezoelectric properties of BaTiO_3_ (BTO)^19–22^ can be enhanced with the ferromagnetic properties of Fe to form a multiferroic material. In fact, previous studies were able to achieve multiferroic films by embedding Fe nanoparticles within a BTO matrix or by growing BTO/Fe multilayers.^23–25^

Driven by the prior studies, the promising applications, as well as the need for integration on fabrication-friendly substrates, a multiferroic vertically aligned nanocomposite (VAN) integrated on Si substrates is explored. This alternative VAN morphology introduces new possibilities compared to the nanoparticle and multilayer techniques, including (1) the additional biaxial interface strain to tune physical properties^26^ and (2) integration on silicon for cost-effective device applications. Recent advancements using the VAN platform beyond the conventional oxide–oxide systems have presented great potential in the growth of self-assembled metal nanopillars in an oxide matrix, such as plasmonic Au nanopillars in a BTO matrix, magnetic Co pillars in BaZrO_3_, and others.^27–30^ It is noted that most of the VAN demonstrations have been focused on single crystal oxide substrates with limited success of oxide–oxide VAN growth on Si substrates,^31,32^ and even fewer reports of oxide–metal VANs on Si.^28^

**Fig. 1:**
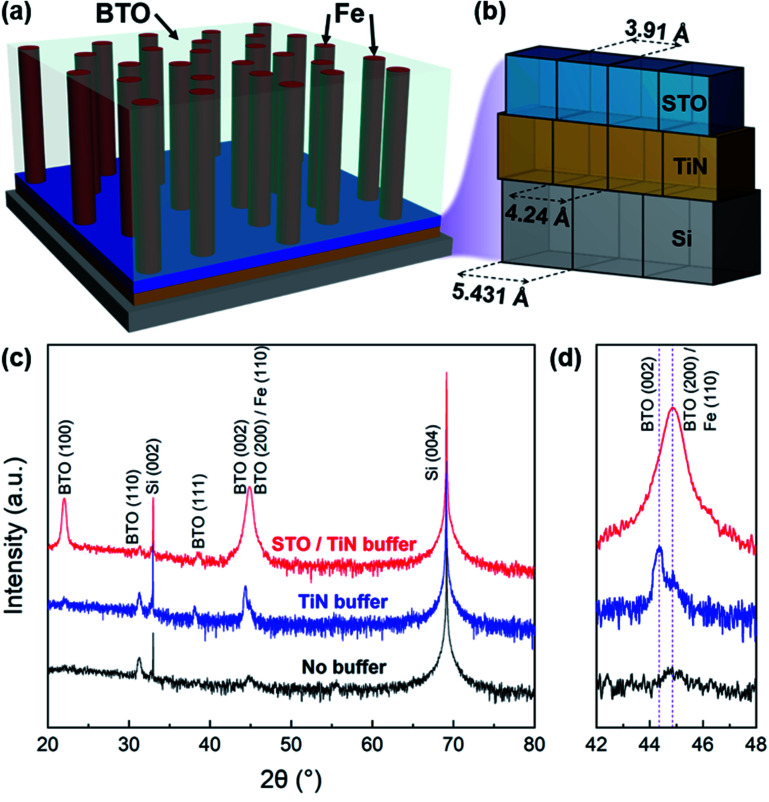
(a) 3D schematic of the BTO–Fe film, buffer layer, and Si substrate stack and (b) lattice/domain match between the TiN and STO buffers and Si substrate. (c) *θ*–2*θ* XRD patterns of BTO–Fe thin films grown on various buffers, with a (d) local scan centered around the BTO (002) peak to distinguish the different phases and orientations.

In this work, a multiferroic BaTiO_3_–Fe (BTO–Fe) VAN system is demonstrated on buffered Si substrates using pulsed laser deposition. The crystallinity and the physical properties are tuned for BTO–Fe films grown on two different buffer configurations: an STO/TiN bilayer buffer and a single layer TiN buffer. These two samples are also compared to a BTO–Fe film grown under the same conditions, but without any buffer. A 3D schematic representing the substrate, buffer layer stack, and proposed VAN system with Fe nanopillars embedded in a BTO matrix is shown in Fig. 1(a). TiN serves as an excellent buffer layer material due to its thermal stability and mechanical integrity. Despite its large lattice mismatch with Si (*f*_TiN on Si_ ≈ 24%), TiN is capable of cube-on-cube growth on Si (001) through domain matching epitaxy, where four TiN lattices match well with three Si lattices,^33^ as illustrated in Fig. 1(b). STO is implemented as an additional buffer layer to further facilitate the growth of the BTO matrix, since its lattice parameter (*a*_STO_ = 3.905 Å) is close to that of BTO (*a*_BTO_ = 3.992 Å). The morphology and physical properties of the BTO–Fe multiferroic VAN thin films are explored using a detailed microstructural characterization, coupled with ferromagnetic, ferroelectric, and optical measurements.

## Results and discussion

The crystallinity and phases of the BTO–Fe target and as-deposited thin films on various buffer stacks were initially characterized by X-ray diffraction (XRD). A *θ*–2*θ* pattern of the BTO–Fe target, shown in Fig. S1,† exhibits polycrystalline BTO, Fe, and Fe_2_O_3_ phases, as expected for a sintered bulk sample. Laser ablation of the target onto Si substrates with different buffer configurations resulted in BTO–Fe thin films with varying crystallinity. XRD *θ*–2*θ* patterns of BTO–Fe films grown on bare Si (no buffer), on a TiN buffer only, and on an STO/TiN buffer stack are compared in Fig. 1(c). Typically, it is difficult to grow epitaxial BTO films directly on Si substrates due to a large lattice mismatch (*f*_BTO on Si_ ≈ 31%) with Si as well as the amorphous SiO_2_ layer at the Si surface. Here, the BTO–Fe film grown without a buffer exhibits relatively small BTO (110) and (002) diffraction peak intensities. For the BTO–Fe film grown on a TiN buffer, the crystallinity of the BTO phase is noticeably improved. Specifically, the intensity of BTO (002) increases dramatically, suggesting improved BTO (00*l*) texturing on the TiN buffer compared to that without any buffer. The *θ*–2*θ* patterns of the BTO–Fe sample grown on an STO/TiN buffer stack show dominant BTO (*h*00) peaks, indicating highly textured growth of the BTO–Fe films and further validating the crucial role of the buffer stack.

Local area *θ*–2*θ* patterns around the BTO (002) peaks are enlarged in Fig. 1(d) and offer additional insights about the crystallinity and added phases of the BTO–Fe thin films. There are two distinct diffraction peak locations for the films grown on buffers. The peak located around 44.35° corresponds to BTO (002), while the peaks centered near 44.85° are likely an overlap of BTO (200) and Fe (110). The difference between BTO (002) and (200) arises from the tetragonal nature of BTO and has previously been reported for bulk and thin film samples.^34,35^ Specifically, the BTO (002) diffraction peak occurs when BTO domains are oriented along the *c*-axis, while the BTO (200) peak is observed from the *a*-axis domains. For the BTO–Fe film grown on the TiN buffer only, the longer *c*-axis domains of BTO (*c*_BTO_ = 4.038 Å) seek to minimize strain with the even larger domains of TiN (*a*_TiN_ = 4.24 Å), resulting in a larger BTO (002) peak compared to that of BTO (200). However, when the BTO–Fe film is grown on the STO/TiN buffer stack, BTO prefers the *a*-axis orientation due to the smaller lattice parameter of STO (*a*_STO_ = 3.91 Å) compared to TiN, as evidenced by the high peak intensity of BTO (200) for this sample. The improved crystallinity of both BTO and Fe phases highlights the critical role of the buffer stack in forming highly textured BTO–Fe films.

**Fig. 2:**
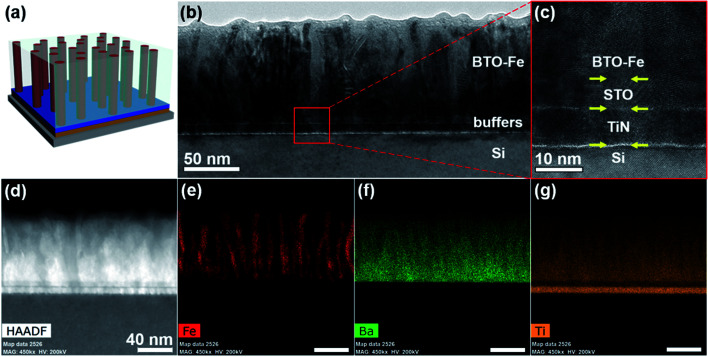
(a) 3D schematic depicting the microstructure of the BTO–Fe film. (b) Cross-section TEM image of the BTO–Fe film grown on a STO/TiN buffer stack with the Fe nanopillars embedded within the BTO matrix. (c) High-magnification TEM image showing the interfaces between the film, buffer layers, and substrate. (d) Cross-section HAADF image of the film, buffer stack, and substrate with EDS elemental maps of (e) Fe, (f) Ba, and (g) Ti.

Microstructural characterization of the BTO–Fe nanocomposite thin film grown on the STO/TiN buffer stack is presented in Fig. 2. A 3D schematic representing the BTO–Fe film and buffer stack is shown in Fig. 2(a). Fig. 2(b) shows a low-magnification cross-sectional TEM image containing the Si substrate, buffer stack, and the BTO–Fe film. The STO and TiN buffer layers are each approximately 10 nm thick and the film, which is roughly 80 nm thick, also reveals vertically aligned dark-contrast Fe nanopillars within the bright-contrast BTO matrix. In Fig. 2(c), a high-resolution TEM image emphasizes the various interfaces present in the sample. The interface between the STO and TiN layers is clean and well-defined, however, the contrast between the BTO matrix and STO buffer is not as apparent, likely due to the similar elemental composition and structure.

To clearly examine the distribution of the elements, scanning transmission electron microscopy (STEM) under high-angle annular dark-field (HAADF) mode coupled with electron-dispersive X-ray spectroscopy (EDS) elemental mappings of Fe, Ba, and Ti were obtained and are displayed in Fig. 2(d–g), respectively. The Fe nanopillars are clearly separated from the oxide matrix and form nanopillars which are dispersed throughout the sample. For example, Fe-rich regions in Fig. 2(e) correspond well with the deficient elements from the BTO matrix, such as Ba and Ti in Fig. 2(f) and (g), respectively, further indicating the phase separation of metallic Fe nanopillars and BTO oxide matrix. These results are also in good agreement with the above XRD *θ*–2*θ* patterns. Additionally, the Ba- and Ti-rich layers which are evident in Fig. 2(f) and (g) further confirm the presence of the STO/TiN buffer stack.

By growing BTO–Fe films on various buffer layers, Fe nanopillar geometries can effectively be tuned. As a comparison, an EDS image of the BTO–Fe film grown directly on the Si substrate (no buffer) is shown in Fig. S2.† For this sample, the Fe nanoinclusions are generally smaller, more sparse, and do not form continuous nanopillars as those seen in the BTO–Fe films grown with the STO/TiN buffer stack. Without the appropriate buffer layers, the BTO domains are more randomly oriented and prevent the Fe from growing in a preferred orientation, leading to less-ordered Fe nanostructures. The subsequent magnetic and optical studies highlight the importance of the uniform Fe nanopillars and reveal additional differences between the BTO–Fe films grown on different buffers.

**Fig. 3:**
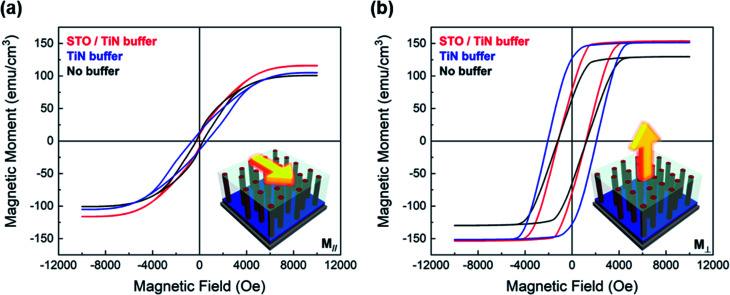
Room-temperature (a) in-plane and (b) out-of-plane magnetic hysteresis loops of the BTO–Fe films deposited on various buffers.

To investigate the ferromagnetic properties of the multiferroic BTO–Fe thin films, both temperature- and field-dependent magnetization measurements were performed. *M*–*T* curves of the films grown on the various buffer layer configurations are presented in Fig. S3† and exhibit a ferromagnetic-like temperature dependence. The in-plane and out-of-plane *M*–*H* responses were also measured for all samples and the results are plotted in Fig. 3(a) and (b), respectively. The hysteresis loops show strong ferromagnetic behavior and obvious anisotropy, with saturation magnetizations (*M*_S_) reaching 155 emu cm^−3^ and 120 emu cm^−3^ for out-of-plane and in-plane measurements, respectively, which is even higher than the *M*_S_ values reported for BTO–Fe films grown on STO substrates.^36^ The magnetic anisotropy originates from the shape anisotropy of the nanostructured Fe pillars, which have an aspect ratio (height/diameter) greater than one. The Fe nanopillars are formed by a self-assembly process, in which the two different phases (BTO and Fe) form columnar domains with a preferred out-of-plane growth orientation. Because of this unique morphology, a stronger out-of-plane magnetization is observed. The out-of-plane magnetic anisotropy of the BTO–Fe thin films becomes even more remarkable when taking into account that the easy axis for Fe is along the in-plane [100] direction, suggesting that the vertical alignment of the Fe nanostructures plays a significant role in the magnetic anisotropy.

Using different buffer layers has a modest, but noticeable, influence on the magnetic properties, namely the saturation magnetization and coercivity. Specifically, the saturation magnetizations (in-plane and out-of-plane) of the BTO–Fe films grown without any buffer are lower those of the other two samples. The *M*_S_ values increase slightly when the BTO–Fe films are grown on buffer layers, likely due to the larger Fe regions and the increased magnetic field required to saturate the domains. The magnetic measurements also seem to suggest that the BTO–Fe films grown on the buffer layers contain larger Fe regions compared with the films grown on the other buffer layers, which is in agreement with the previously discussed microstructural studies. Interestingly, the BTO–Fe film grown on the TiN buffer has the largest coercivity, for both the in-plane and out-of-plane measurements. Coercivity has been shown to increase with increasing Fe particle size until the critical size for a single domain is reached, after which larger Fe particles form multi-domains and coercivity decreases.^37,38^ In this study, the Fe particles in the BTO–Fe film grown without a buffer are smaller than the critical size, and larger in the film grown on the STO/TiN stack, resulting in reduced coercivities compared to the films grown on the TiN buffer. Interestingly, all samples exhibit magnetic anisotropy, including the BTO–Fe film grown directly on Si (without buffer layers). The reason for the similar magnetic properties across all samples is likely due to the similar morphology of the Fe nanopillars in the samples, despite the different film crystallinity and overall epitaxial quality. For example, the EDS image of the BTO–Fe film grown without any buffer layers, previously introduced in Fig. S2,† shows some anisotropic Fe structures, though not as pronounced as those seen in the BTO–Fe film grown on the STO/TiN bilayer buffer. These observations help explain the inherent magnetic anisotropy as well as the slightly reduced saturation magnetization for the sample grown without a buffer. Overall, the magnetization measurements are good indicators of ferromagnetic behavior in the BTO–Fe thin films.

**Fig. 4:**
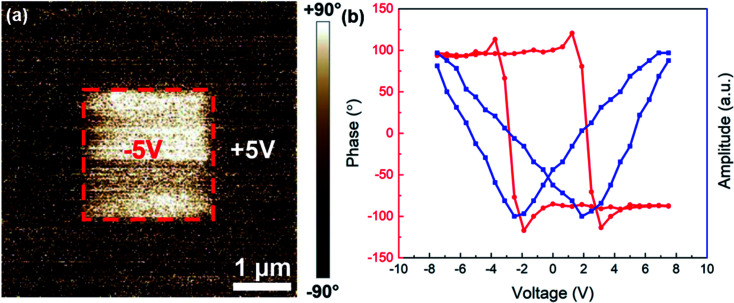
Ferroelectric properties of the BTO–Fe thin film grown on an STO/TiN buffer. (a) PFM out-of-plane phase map after poling with +5 V (dark contrast) and −5 V (bright contrast) and (b) PFM phase and amplitude plots.

The second ferroic order, ferroelectricity, is demonstrated through piezoresponse force microscopy (PFM) measurements, shown in Fig. 4, which present the ferroelectric behavior of the BTO–Fe thin film grown on the STO/TiN bilayer-buffered silicon substrates and further confirm the multiferroic properties of this system. Although all BTO–Fe films grown on the various buffer configurations were measured, only the film grown on the bilayer buffer produced meaningful data. For the other two films (grown directly on Si and with a TiN buffer layer) it is likely that the poor crystallinity of the BTO phase inhibited the electric polarizability, thus no clear ferroelectric effects were observed. This is also supported by the XRD data presented previously. In Fig. 4(a), the out-of-plane phase switching map for the BTO–Fe film was obtained by applying a +5 V bias to the PFM tip and scanning it across a 5 × 5 μm^2^ square box, followed by a 2 × 2 μm^2^ central area scan with a −5 V tip bias. The pronounced contrast in the phase image clearly shows the domain switching, which is characteristic of ferroelectric materials. In addition, the PFM phase and amplitude for the BTO–Fe film grown on the bilayer buffer is presented in Fig. 4(b). The hysteresis phase switching and the butterfly-like amplitude signal, together with the domain switching behavior, unambiguously demonstrate the ferroelectric nature of the BTO–Fe thin films integrated on silicon. Additional measurements performed with a magnetoelectric (ME) bundle reveal room-temperature ME coupling. Similar to the PFM measurement, only the film grown on the STO/TiN bilayer buffer produced significant data. Fig. S4† shows the polarization of the BTO–Fe thin film grown on the STO/TiN bilayer buffer as a function of the applied magnetic field. An ME charge coefficient of *α* = 2.07 × 10^−4^ μC cm^−2^ Oe^−1^ was obtained from the slope of the linear fit, represented by the black line. The more practical ME voltage coefficient *α*_ME_ was determined using the relationship *α* = *α*_ME_*ε*_0_*ε*_r_, where *ε*_0_ and *ε*_r_ represent the vacuum and relative permittivities of the film, respectively. The relative permittivity of a BTO thin film at room temperature^39^ was used for the calculation, yielding an ME voltage coefficient of *α*_ME_ = 4.68 V cm^−1^ Oe^−1^, which is comparable with other BTO-based two-phase studies.^40,41^

**Fig. 5:**
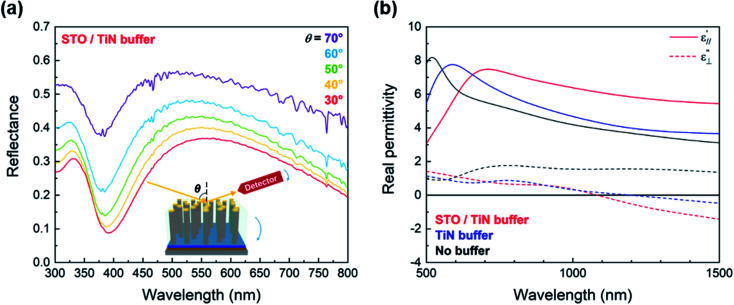
(a) Experimental reflectance (*R*%) spectra of the BTO–Fe film deposited on a STO/TiN buffer stack and (b) in-plane 
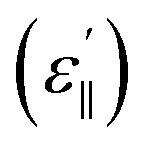
 and out-of-plane 
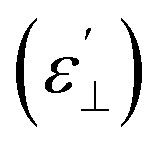
 components of the real permittivity derived from ellipsometric measurements of the BTO–Fe films grown on various buffers.

Fascinating optical properties in pure BTO and BTO nanocomposite thin films have previously been reported, including large Pockel's coefficients and hyperbolic dispersion.^27,28,34,42^ Since the microstructural characterization and magnetic properties revealed highly anisotropic features arising from the Fe nanopillars, the optical properties of the BTO–Fe thin films integrated on Si were also investigated in this study. Angular-dependent reflectance (*R*%) measurements were performed at various incident angles (*e.g.*, 30°, 40°, 50°, 60° and 70°) using a spectroscopic ellipsometer. The measured reflectance spectra for the BTO–Fe film grown on the STO/TiN buffer stack are shown in Fig. 5(a), with the inset showing a schematic illustration of the measurement. The shift in peak reflectance for various incident light angles is one indicator of anisotropic behavior in the BTO–Fe film, likely due to the Fe nanopillars. This shift is a phenomenon which has also been observed in other oxide–metal nanocomposite thin films.^27,28^ The permittivity values of the BTO–Fe films grown on various buffer configurations were also explored using the ellipsometric Psi (*Ψ*) and Delta (*Δ*) data from incident light angles of 55°, 65°, and 75°. In-plane and out-of-plane real permittivity values were derived separately using a uniaxial anisotropy model and are plotted in Fig. 5(b). For all films, the in-plane 
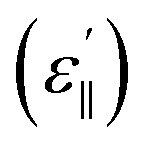
 components are clearly different from the out-of-plane 
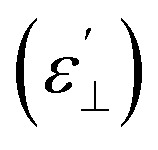
 components, which is additional evidence of optical anisotropy in the BTO–Fe films. While the values of 
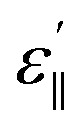
 are positive throughout the 500 nm to 1500 nm range for all the films, the values of 
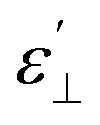
 for the BTO–Fe films grown on the bilayer buffer and TiN buffer become negative around 1100 nm and 1200 nm, respectively. The negative permittivities are characteristic of a hyperbolic metamaterial and can be explained by the presence of metallic Fe nanopillars embedded within the dielectric BTO matrix.

Overall, the demonstration of BTO–Fe thin films on silicon using various buffer layer configurations presents an effective approach for integrating room-temperature multiferroic materials on silicon. First, besides oxide–metal nanocomposites, other complex nanocomposite systems such as oxide–oxide and multi-phase nanocomposite systems can also be effectively integrated, which allows great flexibility in material selections and integration towards multiferroics on silicon. Second, tunability achieved by varying the buffer layers can result in tailorable ferromagnetic and ferroelectric properties within the thin films. Therefore, a novel functional material system coupled with a simple fabrication method could bring tremendous possibilities in the design and fabrication of the nanoscale devices on silicon.

## Conclusion

Self-assembled multiferroic BTO–Fe VAN thin films have been successfully integrated on Si (001) substrates using a PLD technique. Detailed microstructural characterizations including XRD, TEM, and EDS demonstrate the critical role of buffer layers in the epitaxial growth and physical properties of BTO–Fe films. Overall, the films grown on the STO/TiN buffer stack exhibit excellent crystallinity and enhanced multiferroic and optical properties, including ferromagnetic anisotropy and hyperbolic dispersion. The growth of a highly anisotropic BTO–Fe thin film integrated on Si presents great opportunities in the design and fabrication of multiferroic devices for sensing, data storage, and energy harvesting.

## Experimental

A composite BaTiO_3_–Fe target was prepared by mixing BaTiO_3_ and Fe powders (1 : 1 molar ratio), which were then pressed and sintered with inflowing Ar/H_2_. TiN and SrTiO_3_ buffer layers were deposited using commercially obtained targets. The buffer layers and BTO–Fe films were grown on Si (001) substrates at a temperature of 750 °C and under high vacuum (1.0 × 10^−6^ mTorr) by a pulsed laser deposition (PLD) technique using a KrF excimer laser (*λ* = 248 nm) with a laser fluence of 1.9 J cm^−2^. After deposition, the samples were cooled to room temperature in high vacuum. The microstructure and phase composition of the deposited thin films were characterized using X-ray diffraction (XRD, Panalytical Empyrean), transmission electron microscopy (TEM, FEI Talos F200X), scanning transmission electron microscopy (STEM) and electron-dispersive X-ray spectroscopy (EDS). In-plane and out-of-plane magnetic properties were studied in a superconducting quantum interference device (Quantum Design MPMS3). The piezoresponse force microscopy (PFM) measurement was conducted with a Bruker Icon atomic force microscope. Magnetoelectric coupling was measured using a magnetoelectric bundle from Radiant Technologies, Inc. Reflectance spectra and permittivities were obtained with a spectroscopic ellipsometer (J.A. Woollam RC2). The optical permittivity was calculated using appropriate models on CompleteEASE software.

## Conflicts of interest

There are no conflicts to declare.

## Supplementary Material

NA-002-D0NA00405G-s001
