# Supplementary material for: Integration of highly anisotropic multiferroic BaTiO3–Fe nanocomposite thin films on Si towards device applications
Source: Nanoscale Adv. 2020 Jul 21;2(9):4172–8. doi: 10.1039/d0na00405g (PMC9417584; doi:10.1039/d0na00405g)
Supplement: NA-002-D0NA00405G-s001 [file NA-002-D0NA00405G-s001.pdf]

Electronic Supplementary Material (ESI) for Nanoscale Advances.
This journal is © The Royal Society of Chemistry 2020

Supporting Information

Integration of highly anisotropic multiferroic $BaTiO_3$-Fe nanocomposite thin films on Si towards device applications

Matias Kalaswad,[1] Bruce Zhang,[1] Xuejing Wang,[2] Han Wang,[2] Xingyao Gao,[2] and Haiyan Wang[1,2,*]

[1]School of Electrical and Computer Engineering, Purdue University, West Lafayette, Indiana 47907, USA

[2]School of Materials Engineering, Purdue University, West Lafayette, Indiana 47907, USA

Keywords: oxide-metal nanocomposites, room temperature multiferroics, hyperbolic dispersion, silicon integration

[*]Author to whom correspondence should be addressed: hwang00@purdue.edu

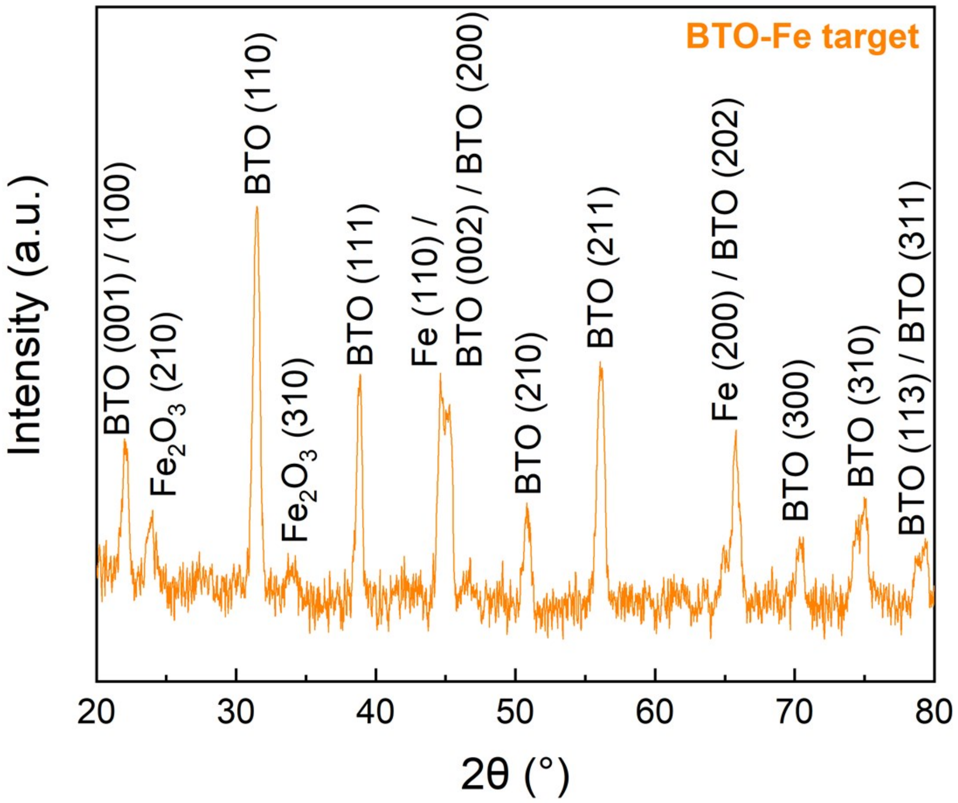

Figure S1. XRD *θ-2θ* pattern of the BTO-Fe target showing polycrystalline BTO, Fe, and Fe_2O_3 phases.

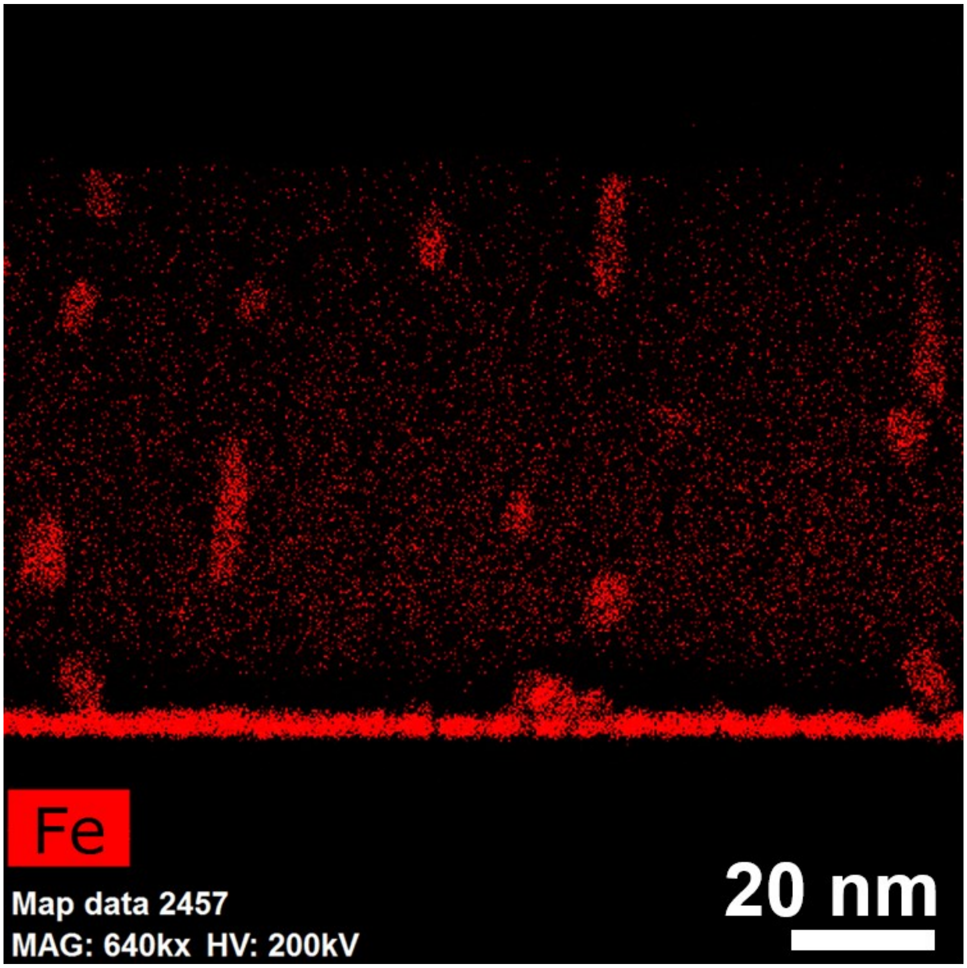

Figure S2. EDS elemental map of the BTO-Fe film grown directly on the Si (001) substrate, without buffer layer.

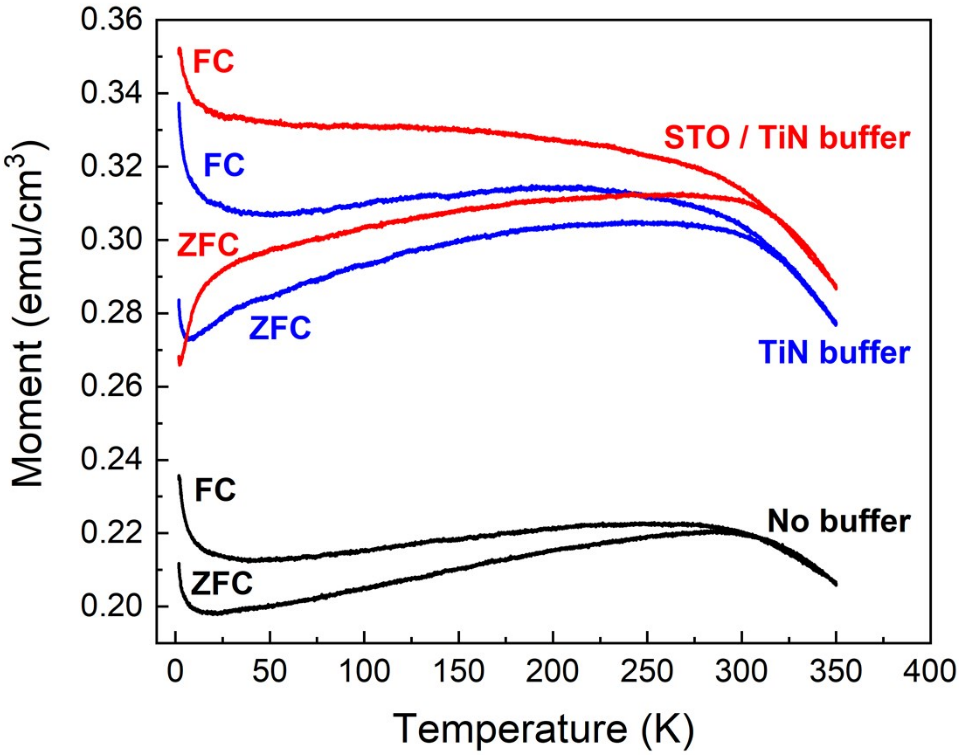

Figure S3. Field cooling (FC) and zero-field cooling (ZFC) *M*-*T* curves of the BTO-Fe thin films grown on various buffer layer configurations.

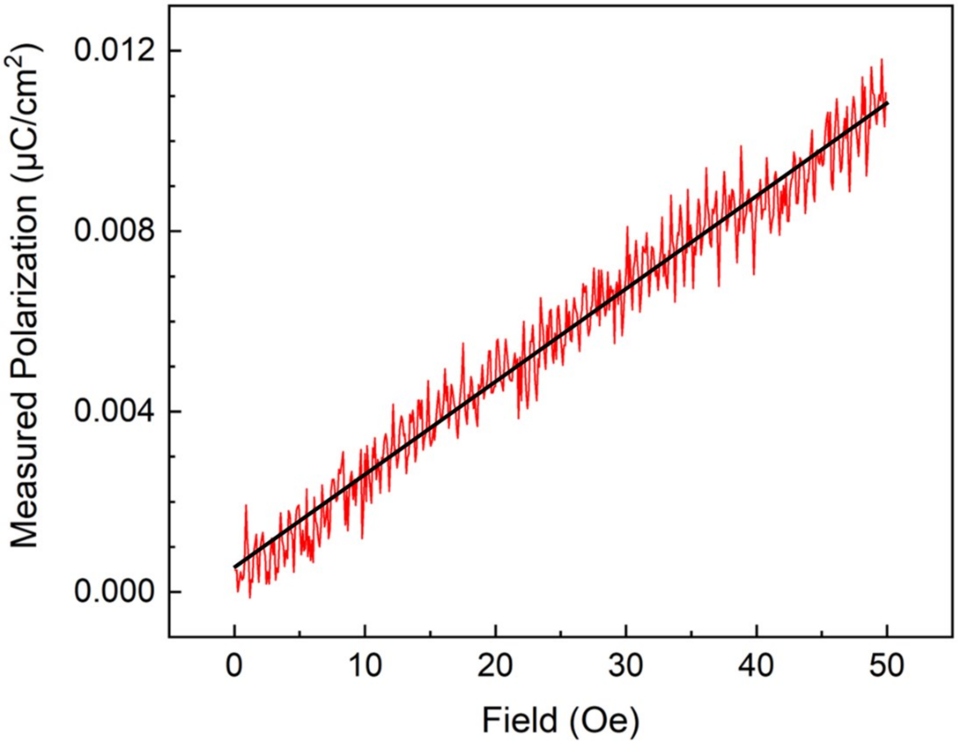

Figure S4. Room-temperature polarization as a function of magnetic field of the BTO-Fe nanocomposite thin film grown on a bilayer-buffered (STO / TiN) silicon substrate. The black line represents a linear fit of the data.
